# Fabrication of Polycrystalline Zeolitic Imidazolate Framework Membranes by a Vapor-Phase Seeding Method

**DOI:** 10.3390/membranes13090782

**Published:** 2023-09-07

**Authors:** Zhiqin Qiang, Zihao Yi, Jun-Wei Wang, Rahul Sampat Khandge, Xiaoli Ma

**Affiliations:** Department of Materials Science and Engineering, University of Wisconsin—Milwaukee, Milwaukee, WI 53201, USA; zqiang@uwm.edu (Z.Q.); zihaoyi@uwm.edu (Z.Y.); wjw@ustc.edu.cn (J.-W.W.); rkhandge@uwm.edu (R.S.K.)

**Keywords:** membrane formation, ZIF membrane, vapor-phase synthesis, atomic layer deposition, propylene/propane separation

## Abstract

The reliable fabrication of polycrystalline zeolitic imidazolate framework (ZIF) membranes continues to pose challenges for their industrial applications. Here, we present a vapor-phase seeding approach that integrates atomic layer deposition (ALD) with ligand vapor treatment to synthesize ZIF membranes with high propylene/propane separation performance. This method began with depositing a ZnO coating onto the support surface via ALD. The support underwent treatment with 2-methylimidazole vapor to transform ZnO to ZIF-8, forming the seed layer. Subsequent secondary growth was employed at near-room temperature, allowing the seeds to grow into a continuous membrane. ZIF-8 membranes made on macroporous ceramic support by this method consistently demonstrated propylene permeances above 1 × 10^−8^ mol Pa^−1^ m^−2^ s^−1^ and a propylene/propane separation factor exceeding 50. Moreover, we demonstrated the effectiveness of the vapor-phase seeding method in producing the ZIF-67 membrane.

## 1. Introduction

Olefin/paraffin separation is in high demand in the petrochemical industry, traditionally performed via energy-intensive cryogenic distillation systems on a large scale [1]. Membrane technology can be more energy-efficient for olefin/paraffin separation [2]. Among the various membrane materials studied, polycrystalline ZIF-8 membranes exhibit exceptional performance for propylene/propane (C3) separation because the diffusivities of propylene and propane within the ZIF-8 framework differ by over two orders of magnitude [3,4,5,6,7]. However, the overall selectivity of the membrane is highly sensitive to any defects formed in the polycrystalline membrane layer. This challenge becomes even more pronounced when reducing the membrane thickness to enhance gas permeances, as eliminating non-selective defects becomes increasingly difficult. Therefore, significant research efforts have been directed toward developing improved fabrication methods to create defect-free ZIF-8 membranes with reproducible separation performances.

Two common methods for fabricating ZIF membranes are in situ synthesis and seeded growth [8,9,10,11,12,13]. In the former method, surface functionalization of the support has been employed to facilitate membrane formation [14,15,16,17]. Although in situ synthesis is straightforward, it often produces relatively thick membranes with moderate gas permeances. In comparison, seeded growth can produce thinner membranes, but it often requires the creation of a homogeneous seed layer [18,19,20,21]. Therefore, various seeding techniques have been developed, including spin-coating [22], manual rubbing [23], dip-coating [24,25,26,27], thermal seeding [28], microwave seeding [29,30,31,32,33], vacuum filtration [34], and the use of sacrificial of ZIF-L as the seed layer [35].

The mild synthesis conditions for ZIF-8 formation have also given rise to a variety of innovative membrane fabrication methods, including counter-diffusion synthesis [36,37], metal ion pre-anchored strategy [38], microfluidic processing [39,40], aqueously cathodic deposition [41], current-driven synthesis [42,43], inhibited Ostwald ripening [44], electrophoretic nuclei assembly [45], gel–vapor deposition [46,47], and all-vapor-phase processing [48,49]. For instance, Li et al. prepared ultra-thin ZIF-8 membranes with controllable thickness via a gel–vapor method, which combined sol–gel synthesis and solvent-free vapor deposition [46]. In another work, Ma et al. reported an all-vapor-phase method, named ligand-induced permselectivation (LIPS), that converted ZnO thin films made by atomic layer deposition (ALD) to ZIF-8 membranes through treatment with 2-methylimidazole (2-mIm) vapor [48]. The use of mesoporous γ-alumina support is crucial in this method, as its small mesopores allow the formation of a thin, impermeable ZnO coating prior to ligand vapor treatment. This approach is more cost-effective and sustainable than solution-based methods by eliminating the use of solvents. In addition, the conformal vapor–solid reaction facilitates the creation of thin membranes with high propylene fluxes [48]. Recently, this method has been employed to synthesize high-flux ZIF-8 membranes on anodic alumina oxide (AAO) supports that have mesopores of ~20 nm [50]. However, vapor-phase synthesis has not been extended to macroporous ceramic supports, which could lower the membrane cost.

Here, we developed a vapor-phase seeding method to create a conformal and compact ZIF-8 seed layer that can be easily grown into a continuous membrane on macroporous ceramic support via near-room temperature secondary growth. In the seeding step, ZnO was deposited onto the support using ALD and then converted into ZIF-8 seeds through exposure to 2-mIm vapors. Subsequent secondary growth of these seeds resulted in well-intergrown polycrystalline membranes. In addition, we investigated the impact of the ALD cycle number on membrane structure and properties. Our results show that the vapor-phase seeding approach is highly effective on macroporous ceramic supports, and it can also be employed to fabricate the ZIF-67 membrane. Compared to conventional solution-based seeding methods, vapor-phase seeding can eliminate solvent use for seed synthesis, enhance the adhesion between seeds and support, and produce a more compact seed layer for membrane growth.

## 2. Materials and Methods

### 2.1. Materials

Alumina powders (Baikalox 1.0CR, average particle size of ~0.5 µm) from the Baikowski International Corporation (Charlotte, NC, USA) were used to prepare the macroporous α-alumina supports. Nitric acid (1.0 N standardized solution, Alfa Aesar, Haverhill, MA, USA), zinc nitrate hexahydrate (Zn(NO_3_)_2_·6H_2_O, 98%, Sigma–Aldrich, St. Louis, MO, USA), 2-methylimidazole (C_4_H_6_N_2_, 99%, Sigma–Aldrich, St. Louis, MO, USA), cobalt nitrate hexahydrate (Co(NO_3_)_2_·6H_2_O, ACS reagent, ≥98%, Sigma–Aldrich, St. Louis, MO, USA), methanol (CH_3_OH, ACS reagent, ≥99.8%, Sigma–Aldrich, St. Louis, MO, USA), were used to prepare ZIF membranes.

### 2.2. Preparation of Macroporous Supports

Macroporous α-alumina supports with a diameter of 22 mm and a thickness of 2 mm were prepared by a casting–sintering method reported in the literature [48]. The supports were polished with silicon carbide paper (MicroCut Plain, Buehler, Lake Bluff, IL, USA) and dried at 250 °C for 10 h.

### 2.3. Fabrication of ZIF Membranes by Vapor-Phase Seeding

**Figure 1:**
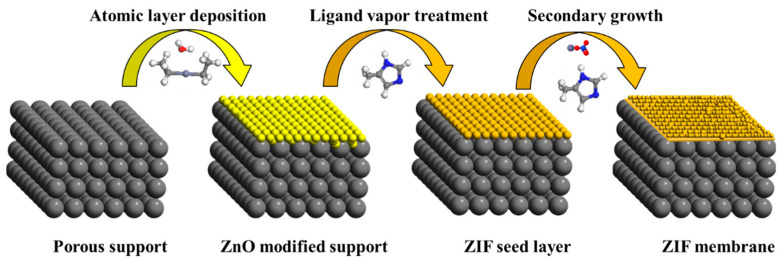
A schematic illustration depicting the procedures for fabricating ZIF membranes using vapor-phase seeding and secondary growth.

ZIF membranes were fabricated using the vapor-phase seeding and secondary growth methods, as shown in Figure 1. GEMStar XT-S/D™ Benchtop Thermal ALD (Arradiance, LLC, Littleton, MA, USA) was used to deposit ZnO on the porous supports at a deposition temperature of 125 °C. The supports were placed horizontally in the center of the ALD chamber, and the system was evacuated for 30 min. During ALD, the gaseous reactants, diethylzinc (DEZ) used as the precursor and H_2_O as the oxidant, were alternately introduced into the reaction chamber. A typical ALD cycle consists of a 15 ms pulse of DEZ, 1 s exposure, 19 s purge with N_2_, 15 ms pulse of H_2_O, 1 s exposure, and 19 s purge with N_2_. The numbers of deposition cycles (10, 20, 30, and 40 cycles) were varied to control the amount of ZnO deposits. The deposition temperature was maintained at 125 °C under all conditions. To transform ZnO into ZIF-8 seeds, The ZnO-modified support was vertically held above 2-methylimidazole (0.2 g) powders at a distance of around 4 cm in a PTFE container and then heated to 125 °C for one day. The 2-methylimidazole vapors were generated to react with ZnO, forming ZIF-8. A temperature of 125 °C was chosen as the treatment temperature because of the effective conversion of ZnO to ZIF-8 under this condition [48,51]. The as-synthesized seeded support was directly used in the next step without further treatment. Then, the ZIF-8 seeded support was placed vertically in a solution containing 2.27 g 2-mIm and 0.11 g Zn (NO_3_)_2_·6H_2_O dissolved in 40 mL of deionized (DI) water, and the system was held at 30 °C for 6 h. After the synthesis, the membrane was rinsed with methanol and dried at room temperature for 1 day before gas permeation measurement and structure characterizations. The ZIF-67 membrane was synthesized by epitaxial growth of the ZIF-8 seed layer. The ZIF-8 seeded support prepared by the vapor-phase seeding method was placed in a solution that contained 0.108 g Co(NO_3_)_2_·6H_2_O and 2.27 g 2-mIm dissolved in 40 mL of DI water and underwent secondary growth at 30 °C for 6 h. 

### 2.4. Gas Permeation Measurements

The gas permeation system with the membrane was evacuated for 2 h before providing feed gas at 1 atm. Permeance was calculated using the pressure increases on the permeate side during gas permeation measurement. For the mixture gas separation test, an equimolar C_3_H_6_/C_3_H_8_ mixture was used as the feed, and Ar was used as the sweep gas. Gas composition in the feed and permeate was measured by gas chromatography (Thermo Scientific TRACE 1300 GC, Waltham, MA, USA) with a flame ionization detector (FID). The separation factor was calculated using the following equation:

aij=yiyjxixj

where $x_{i}\;$ and $x_{j}$ represent the molar fraction of C_3_H_6_ and C_3_H_8_ in the feed, respectively, and $y_{i}$ and $y_{j}$ are their corresponding fractions in the permeate. 

### 2.5. Characterizations

Scanning electron microscope (SEM) images were collected on a Hitachi S4800 field emission SEM coupled with energy dispersive X-ray analysis (EDS). Samples were coated with 5 nm thick iridium in the Emitech K575X Sputter coater (Emitech Ltd., Ashford, Kent, UK) before SEM analysis. X-ray diffraction patterns (XRD) of supports and membranes were obtained using a Bruker D8 Discover A25 diffractometer (Bruker Corporation, Billerica, MA, USA) equipped with a Cu Kα X-ray radiation source (40 kV and 40 mA, λ = 0.154 nm).

## 3. Results and Discussion

### 3.1. Microstructure Evolvement during Membrane Fabrication

**Figure 2:**
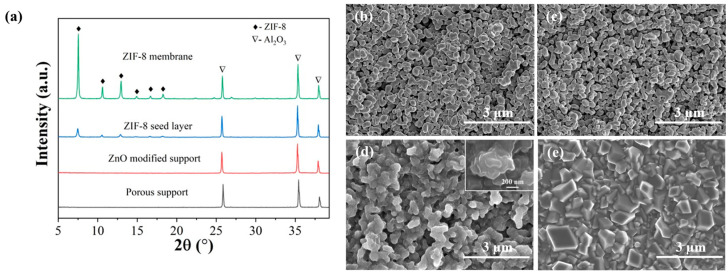
XRD patterns (**a**) and SEM images of pure support (**b**), support modified with 20 cycles ZnO ALD (**c**), ZIF-8 seed layer after ligand vapor treatment (**d**), and ZIF-8 membrane (**e**). Insert in (**d**) shows the higher-magnification image of seeds.

In the vapor-phase seeding process, the support was first coated with ZnO using ALD. Then, the ZnO deposits were transformed into a layer of ZIF-8 seeds by undergoing a vapor-phase treatment with sublimated 2-mIm vapors. Subsequently, the membrane was formed by subjecting the seed layer to secondary growth. During one ALD cycle, the metal precursors (DEZ) react with the hydroxyl groups of α-alumina to produce surface-adsorbed ethylzinc groups, which then react with the oxidant water, forming hydroxylated zinc oxide. As shown in Figure 2a, the ZnO deposited from ALD appears to be amorphous due to the relatively low deposition temperature (125 °C) and the low number of ALD cycles (<50 cycles) used in this study. ZIF-8 XRD peaks were observed in the seeded support after treating the amorphous ZnO with 2-mIm vapor. The increase in peak intensity in the membrane suggests the continued growth of seeds during the secondary growth step. 

SEM was employed to reveal the morphological changes that occurred during membrane synthesis. As shown in Figure 2b, the α-alumina support contains macropores of several hundred nanometers. After coating the support with ZnO via ALD, no morphological changes were detected by high-resolution SEM (Figure 2c), indicating the formation of ultra-thin and conformal ZnO deposits on the surface of Al_2_O_3_ grains. Following ligand vapor treatment, ZIF-8 nanocrystals appeared on the support surface (Figure 2d). The transformation from ZnO to ZIF-8 is accompanied by a ~17 times volume increase due to the different densities of these two materials [52]. This expansive conversion led to closely packed or even partially inter-grown ZIF-8 seeds with small inter-particle voids. These voids were easily eliminated by the continued growth of the ZIF-8 seeds during the near-room temperature secondary growth step. Therefore, the resulting membrane is of high quality and continuous without any visible voids or defects (Figure 2e).

**Figure 3:**
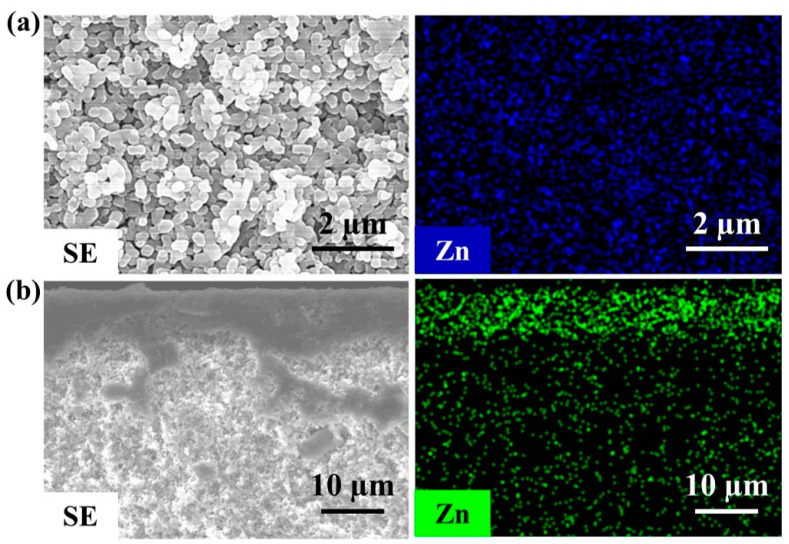
EDS analysis of ZnO ALD modified support (**a**) top view and (**b**) side view.

The ZnO ALD-modified support was further characterized by EDS, which confirmed the presence of Zn (Figure 3a). Cross-section EDS analysis unveiled a penetration depth of ~9 μm for the ZnO deposition within the α-alumina support after 40 cycles ALD (Figure 3b and Figure S1). FTIR and XPS results revealed the presence of Zn–O bonds (Figure S2). These observations, coupled with the XRD results, suggest the amorphous nature of the ZnO deposits.

### 3.2. Effect of ALD Cycle Number

**Figure 4:**
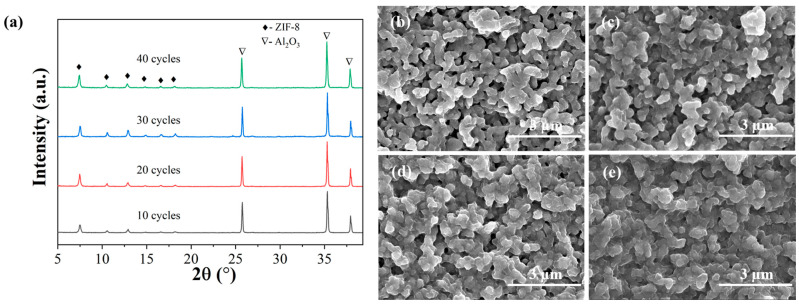
XRD patterns (**a**) and SEM images (**b**–**e**) of ZIF-8 seed layers prepared using different numbers of ALD cycles (10, 20, 30, and 40 cycles).

**Figure 5:**
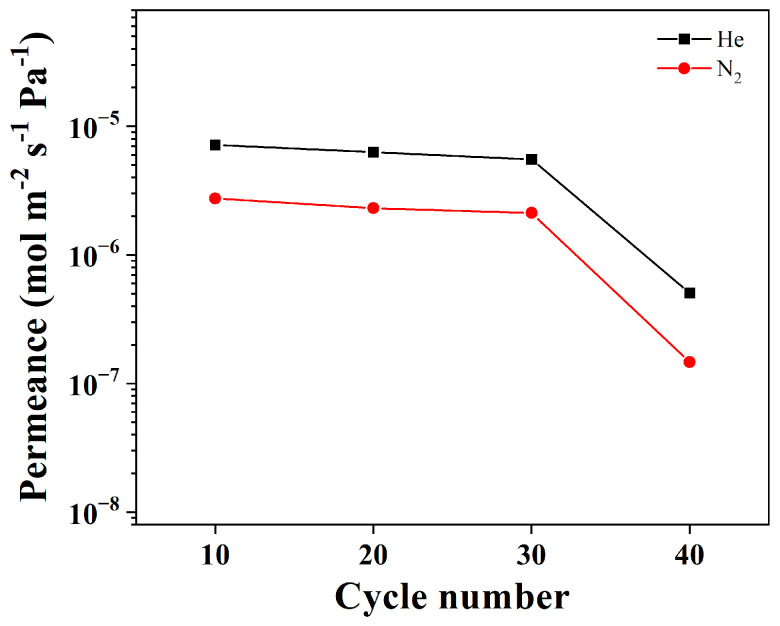
He and N_2_ single-component permeances of ZIF-8 seed layer as a function of the number of ALD cycles (10, 20, 30, and 40 cycles).

A systematic investigation was conducted to understand the impact of the ALD cycle number on membrane structure and properties. The ligand vapor treatment and secondary growth conditions were kept unchanged while varying the ALD parameters. The ALD cycle numbers were varied using a pulse of 15 ms for DEZ and water. The XRD peak intensity of ZIF-8 seed layers increased slightly with ALD cycle numbers (Figure 4). Top-view SEM images in Figure 4 show uniform ZIF-8 seeds under all conditions. With an increase in ALD cycles, the seed layer appeared denser, with the ZIF-8 nanoparticles becoming slightly larger, consistent with the increased intensities in XRD peaks. The single gas permeance of the ALD-modified support was also evaluated. The He/N_2_ ideal selectivity of seeded supports matched that predicted from the Knudsen diffusion, and the gas permeances decreased with increasing ALD cycle numbers (Figure 5). The apparent reduction in gas permeances at 40 cycles could be attributed to the increased penetration depth of ZnO ALD inside the support (Figure S1). Moreover, the seeded supports showed no C3 selectivity.

**Figure 6:**
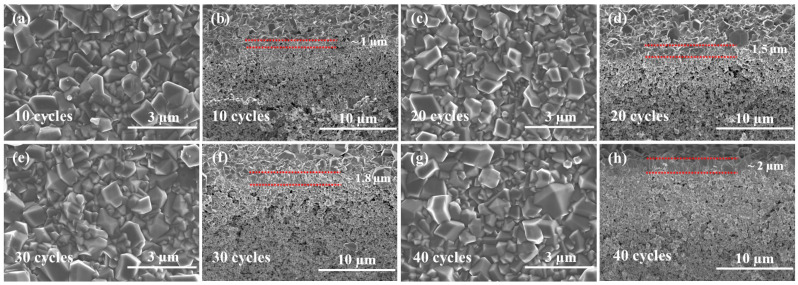
SEM images of ZIF-8 membranes made under different ZnO ALD cycles: (**a**,**b**) 10 cycles, (**c**,**d**) 20 cycles, (**e**,**f**) 30 cycles, and (**g**,**h**) 40 cycles.

**Figure 7:**
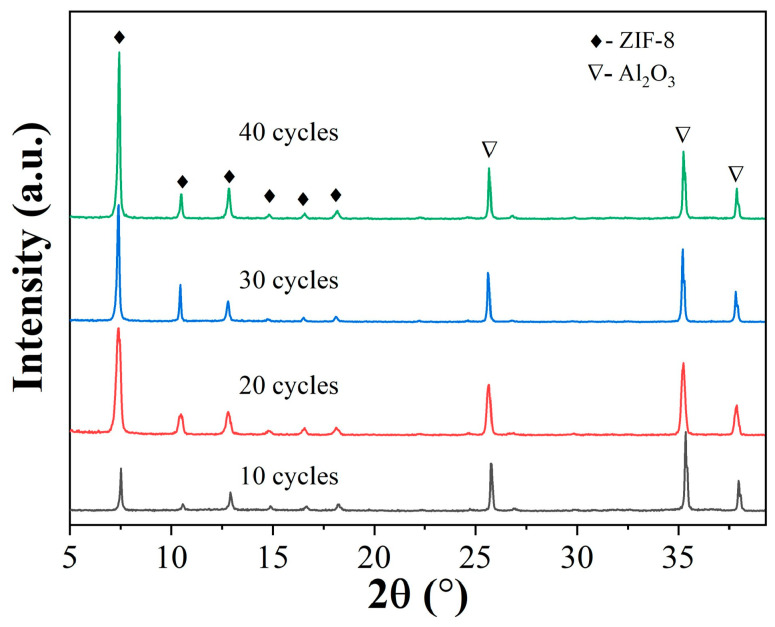
XRD patterns of ZIF-8 membranes made under different ZnO ALD cycles.

Top-view SEM images (Figure 6) show that all membranes are highly continuous, without apparent pinholes or defects. However, cross-sectional SEM images revealed an increase in membrane thickness with cycle numbers. Specifically, the thickness was less than 1 µm at 10 cycles, approximately 1.5 µm at 20 cycles, and 1.5–2 µm at 30 and 40 cycles. ZIF-8 membranes exhibited much stronger XRD peaks than seed layers, with peak intensity increasing with ALD cycle numbers (Figure 7), consistent with the observation from SEM. 

**Figure 8:**
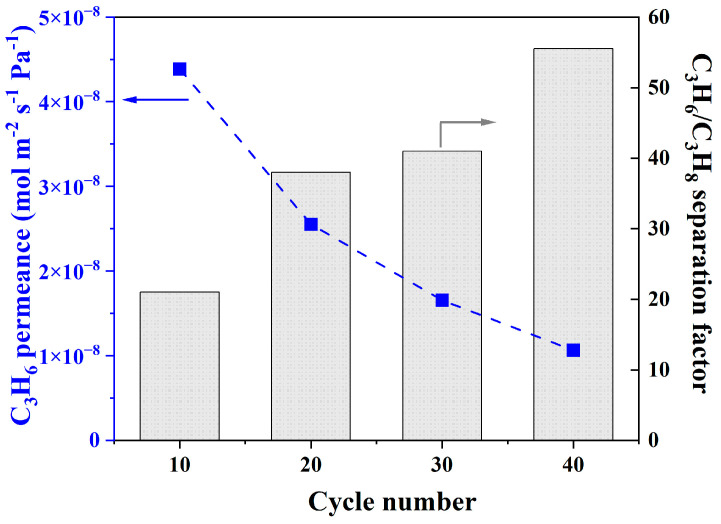
C3 separation performances of ZIF-8 membranes made under different numbers of ALD cycles.

These different thicknesses resulted in varying C3 separation properties. Specifically, propylene permeance decreased, but the C3 separation factor increased with the ALD cycle number (Figure 8). At a 40-cycle ALD, the separation factor reached 55.5 with a propylene permeance of 1.1 × 10^−8^ mol Pa^−1^ m^−2^ s^−1^. On the other hand, it is worth noting that the propylene permeance was not linearly proportional to the inverse of membrane thickness. For instance, compared to the membrane prepared with 10 cycles, the membrane produced using 40 cycles had a propylene permeance that was approximately four times lower despite being only around 100% thicker. This difference can be explained by the different amounts of defects in the membranes and the additional transport resistance caused by ZIFs formed inside the support pores (Figure S3). Overall, these results showed that increasing the ALD cycle number led to more closely packed seeds, which resulted in membranes with enhanced separation factors and good gas permeances.

We synthesized additional membranes to evaluate the reproducibility of the vapor-phase seeding method. As shown in Table 1, these ZIF-8 membranes exhibited propylene permeance ranging from 8 × 10^−9^ to 2.5 × 10^−8^ mol Pa^−1^ m^−2^ s^−1^. Moreover, they exhibited a propylene/propane separation factor surpassing 45, with one membrane reaching a high separation factor of 102. Compared to recently reported polycrystalline ZIF-8 membranes prepared on macroporous α-alumina supports (Figure S4), the overall performance of our membranes ranks at the higher end in terms of the mixed-gas separation factor and propylene permeance. The effect of feed pressure, feed composition, and temperature on membrane performance was also investigated (Figure S5). The membrane exhibited good performance across the varied permeation conditions studied. As feed pressure, temperature, or propylene fraction in the feed increased, separation factors exhibited a slight decline, consistent with trends reported in the literature [5,26,48]. These results have demonstrated that vapor-phase seeding is a reliable and reproducible method for fabricating high-quality ZIF-8 membranes on low-cost macroporous supports. 

A relatively high temperature (~125 °C) was used in our work for the ALD and ligand vapor treatment steps, necessitating the utilization of thermally stable support. Further improving the vapor-phase seeding method to lower the processing temperature would be highly beneficial, potentially enabling membrane fabrication on more cost-effective supports, such as polymer substrates. In addition, extending the vapor-seeding approach to supports with a tubular or hollow fiber geometry is vital for the scale-up of ZIF membranes. 

### 3.3. ZIF-67 Membrane Made by Vapor-Phase Seeding

**Figure 9:**
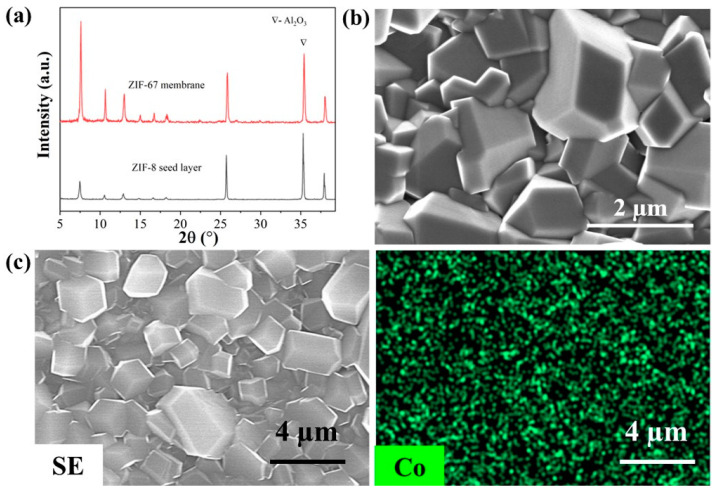
XRD patterns (**a**), SEM image (**b**), and EDS image (**c**) of ZIF-67 membrane prepared on macroporous ceramic support.

We conducted preliminary investigations into the preparation of other ZIF membranes by the epitaxial growth of ZIF-8 seeds created through the vapor-phase seeding approach. Despite using a ZIF-8 seed layer, XRD analysis (Figure 9a) confirmed that the target ZIF-67 phase was successfully formed in the membrane after the heteroepitaxial secondary growth. Top-view SEM images (Figure 9b) show that the ZIF-67 membrane exhibited a high level of continuity and integrity. EDS analysis (Figure 9c) revealed the presence of cobalt elements in the ZIF-67 membrane. The larger crystal size of ZIF-67 in the membrane might be due to the hindered nucleation caused by the cobalt salts, resulting in a reduced number of nucleating centers that allow for the growth of larger crystals [53,54]. The membrane thickness was determined to be ~4 µm from the cross-sectional SEM image (Figure S6). Furthermore, the membrane exhibited a propylene permeance of 4.74 × 10^−9^ mol m^−2^ s^−1^ Pa^−1^ and a separation factor of ~78 for the separation of the propylene/propane binary mixture.

## 4. Conclusions

In conclusion, our study demonstrates the effectiveness of the vapor-phase seeding approach, which relies on ALD and ligand vapor treatment, in creating uniform and compact ZIF-8 seeds that can be readily grown into high-quality ZIF-8 membranes on macroporous supports with high reproducibility. The ALD cycle number was found to influence membrane microstructure strongly, and varying ALD cycle numbers can be used to tune the membrane performance. Additionally, we have demonstrated the feasibility of this approach in synthesizing the polycrystalline ZIF-67 membrane by combining it with epitaxial growth.

## Figures and Tables

**Table 1:** Permeances and propylene/propane separation factors of ZIF-8 membranes fabricated by vapor-phase seeding method.

Membranes	Propylene Permeance (×10^−10^ mol m^−2^ s^−1^ Pa^−1^)	Separation Factor
M1	202.91	64
M2	235.25	48
M3	246.99	45
M4	79.17	52
M5	241.1	102

## Data Availability

The data presented in this study are available upon request from the corresponding author.

## Supplementary Materials

The following supporting information can be downloaded at: https://www.mdpi.com/article/10.3390/membranes13090782/s1, Figure S1. EDS analysis of support modified with 40 cycles ZnO ALD: (**a**) line scan and (**b**) mapping. Figure S2. (**a**) FTIR analysis of support modified with ZnO ALD, (**b**) XPS survey spectra for ZnO ALD modified support ranging from 1100 to 0 eV, and highlighted XPS spectra for (**c**) Zn 2p and (**d**) Zn (LMMM). Figure S3. EDS analysis of the cross-section of ZIF-8 membrane. Figure S4. Comparison of the C_3_H_6_/C_3_H_8_ separation performance of the ZIF-8 membranes made in this work with other ZIF-8 membranes fabricated on macroporous α-alumina supports reported in the literature [3,5,8,27,29,30,36,55,56,57,58,59,60,61,62,63,64,65,66]. Figure S5. The binary C_3_H_6_/C_3_H_8_ separation performance of ZIF-8 membrane as a function of (**a**) feed pressure, (**b**) temperature, and (**c**) C_3_H_6_ fraction in the feed. Figure S6. (**a**) SEM image and (**b**) EDS analysis of ZIF-67 membrane.
