# Supplementary material for: Fabrication of Polycrystalline Zeolitic Imidazolate Framework Membranes by a Vapor-Phase Seeding Method
Source: Membranes (Basel). 2023 Sep 7;13(9):782. doi: 10.3390/membranes13090782 (PMC10538002; doi:10.3390/membranes13090782)
Supplement: Supplementary file 1 [file membranes-13-00782-s001.zip › membranes-2568547-supplementary.pdf]

Supplementary Materials

Fabrication of polycrystalline zeolitic imidazolate framework membranes by a vapor-phase seeding method

Zhiqin Qiang, Zihao Yi, Junwei Wang, Rahul Sampat Khandge, Xiaoli Ma*

Department of Materials Science and Engineering, University of Wisconsin–Milwaukee, Milwaukee, WI, 53201, USA
*Correspondence: ma26@uwm.edu

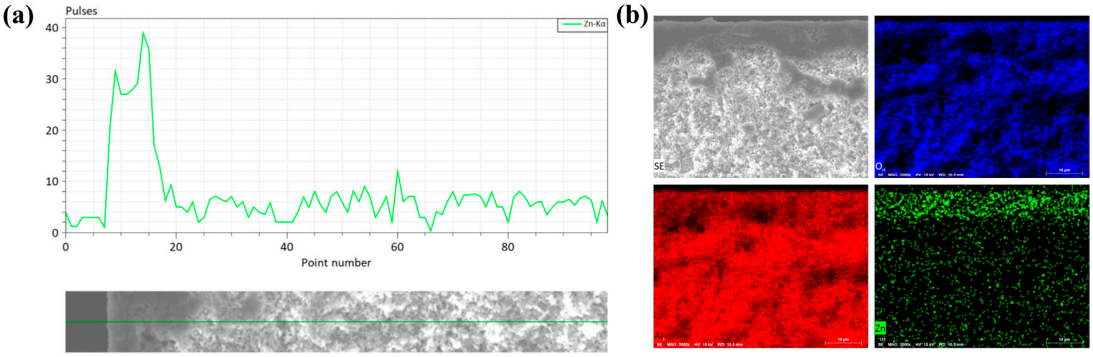

Figure S1. EDS analysis of support modified with 40 cycles ZnO ALD: (a) line scan and (b) mapping.

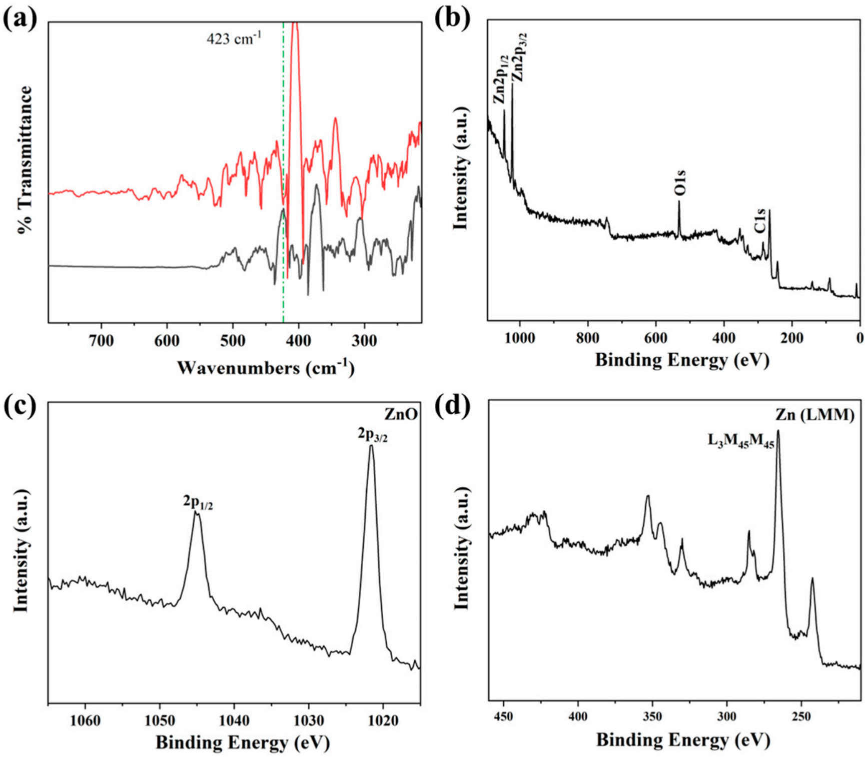

Figure S2. (a) FTIR analysis of support modified with ZnO ALD, (b) XPS survey spectra for ZnO ALD modified support ranging from 1100 to 0 eV, and highlighted XPS spectra for (c) Zn 2p and (d) Zn (LMMM).

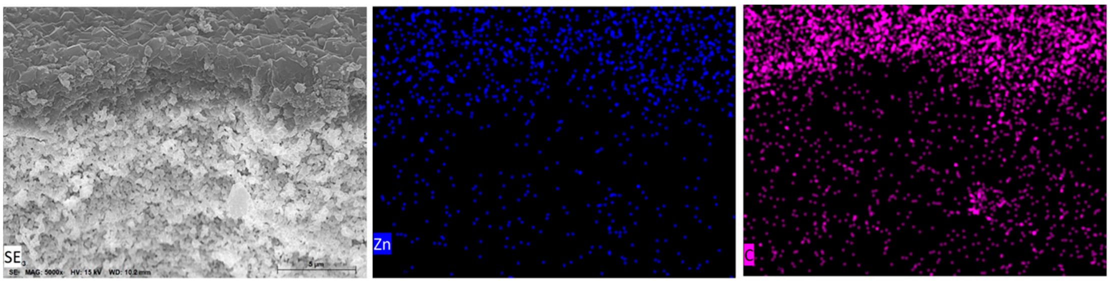

Figure S3. EDS analysis of the cross-section of ZIF-8 membrane.

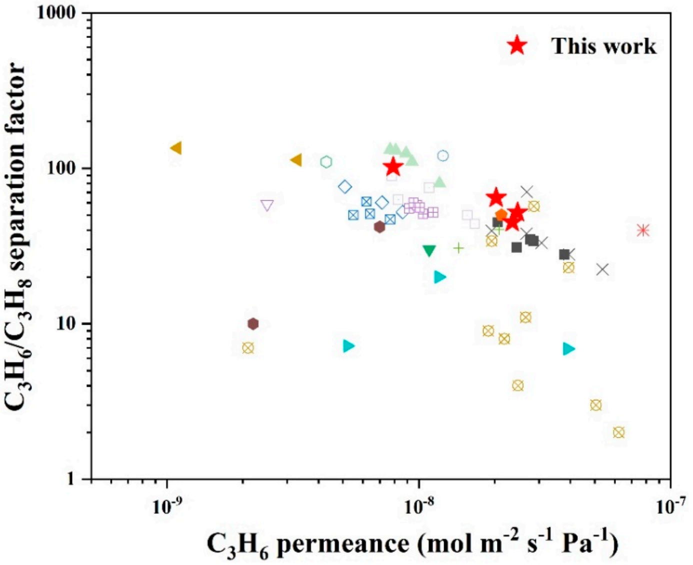

Figure S4. Comparison of the C_3H_6/C_3H_8 separation performance of the ZIF-8 membranes made in this work with other ZIF-8 membranes fabricated on macroporous α-alumina supports reported in the literature [1-19].

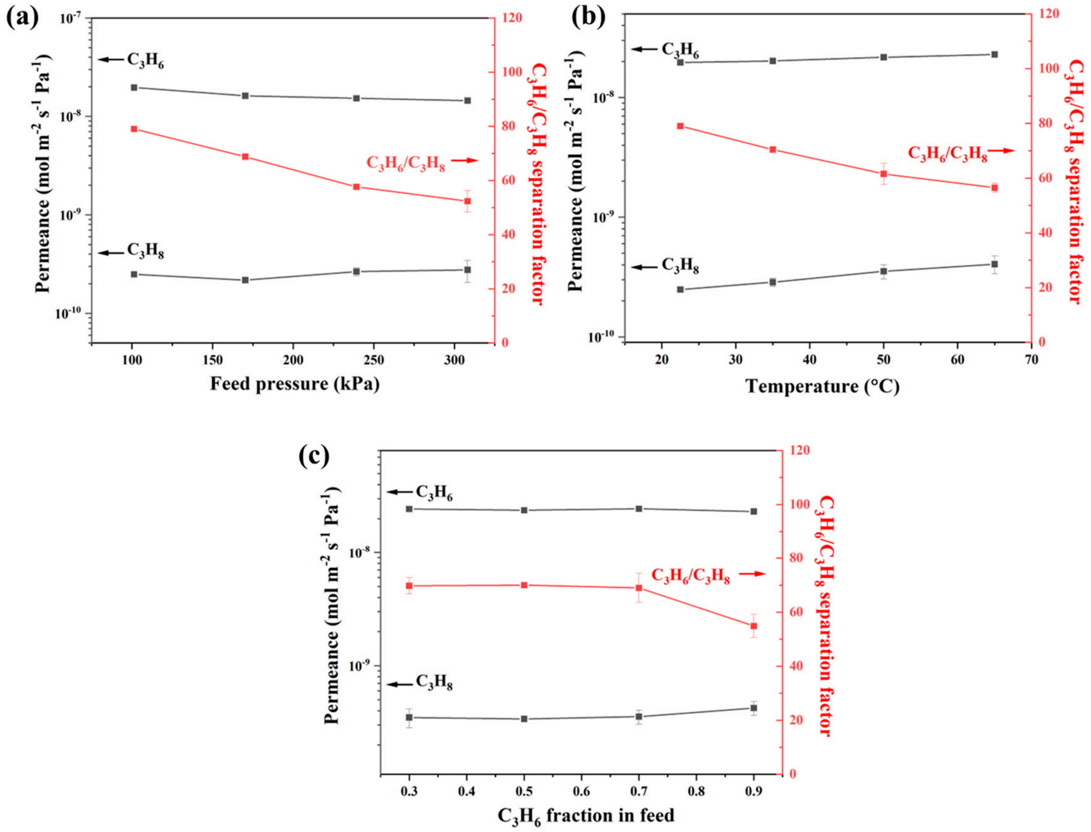

Figure S5. The binary C_3H_6/C_3H_8 separation performance of ZIF-8 membrane as a function of (a) feed pressure, (b) temperature, and (c) C_3H_6 fraction in the feed.

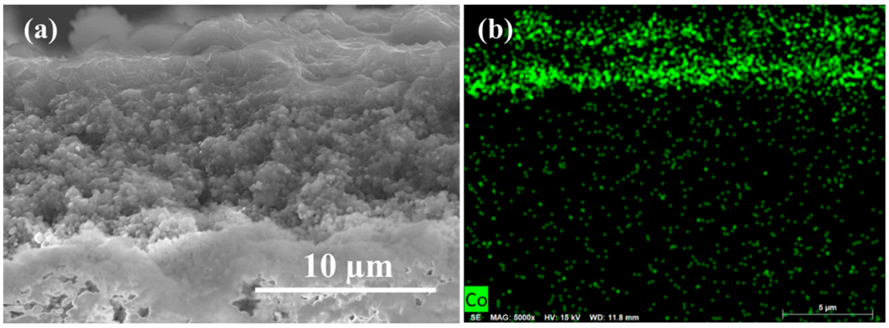

Figure S6. (a) SEM image and (b) EDS analysis of ZIF-67 membrane.

References

1. Pan, Y.; Li, T.; Lestari, G.; Lai, Z. Effective separation of propylene/propane binary mixtures by ZIF-8 membranes. *J. Membr. Sci.* **2012**, *390*, 93-98.

2. Pan, Y.; Liu, W.; Zhao, Y.; Wang, C.; Lai, Z. Improved ZIF-8 membrane: Effect of activation procedure and determination of diffusivities of light hydrocarbons. *J. Membr. Sci.* **2015**, *493*, 88-96.

3. Yu, J.; Pan, Y.; Wang, C.; Lai, Z. ZIF-8 membranes with improved reproducibility fabricated from sputter-coated ZnO/alumina supports. *Chem. Eng. Sci.* **2016**, *141*, 119-124.

4. Liu, D.; Ma, X.; Xi, H.; Lin, Y. Gas transport properties and propylene/propane separation characteristics of ZIF-8 membranes. *J. Membr. Sci.* **2014**, *451*, 85-93.

5. Hara, N.; Yoshimune, M.; Negishi, H.; Haraya, K.; Hara, S.; Yamaguchi, T. Diffusive separation of propylene/propane with ZIF-8 membranes. *J. Membr. Sci.* **2014**, *450*, 215-223.

6. Hara, N.; Yoshimune, M.; Negishi, H.; Haraya, K.; Hara, S.; Yamaguchi, T. ZIF-8 membranes prepared at miscible and immiscible liquid–liquid interfaces. *Microporous Mesoporous Mater.* **2015**, *206*, 75-80.

7. Hara, N.; Yoshimune, M.; Negishi, H.; Haraya, K.; Hara, S.; Yamaguchi, T. Effect of temperature on synthesis of ZIF-8 membranes for propylene/propane separation by counter diffusion method. *Journal of the Japan Petroleum Institute* **2015**, *58*, 237-244.

8. Hara, N.; Yoshimune, M.; Negishi, H.; Haraya, K.; Hara, S.; Yamaguchi, T. Effect of solution concentration on structure and permeation properties of ZIF-8 membranes for propylene/propane separation. *J. Chem. Eng. Jpn.* **2018**, *49*, 97-103.

9. Kwon, H.T.; Jeong, H.-K.; Lee, A.S.; An, H.S.; Lee, J.S. Heteroepitaxially grown zeolitic imidazolate framework membranes with unprecedented propylene/propane separation performances. *J. Am. Chem. Soc.* **2015**, *137*, 12304-12311.

10. Kwon, H.T.; Jeong, H.-K. In situ synthesis of thin zeolitic–imidazolate framework ZIF-8 membranes exhibiting exceptionally high propylene/propane separation. *J. Am. Chem. Soc.* **2013**, *135*, 10763-10768.

11. Kwon, H.T.; Jeong, H.-K.; Lee, A.S.; An, H.S.; Lee, T.; Jang, E.; Lee, J.S.; Choi, J. Defect-induced ripening of zeolitic-imidazolate framework ZIF-8 and its implication to vapor-phase membrane synthesis. *Chem. Commun.* **2016**, *52*, 11669-11672.

12. Kwon, H.T.; Jeong, H.-K. Highly propylene-selective supported zeolite-imidazolate framework (ZIF-8) membranes synthesized by rapid microwave-assisted seeding and secondary growth. *Chem. Commun.* **2013**, *49*, 3854-3856.

13. Kwon, H.T.; Jeong, H.-K. Improving propylene/propane separation performance of Zeolitic-Imidazolate framework ZIF-8 Membranes. *Chem. Eng. Sci.* **2015**, *124*, 20-26.

14. Lee, M.J.; Kwon, H.T.; Jeong, H.K. High-flux zeolitic imidazolate framework membranes for propylene/propane separation by postsynthetic linker exchange. *Angew. Chem. Int. Ed.* **2018**, *57*, 156-161.

15. Tran, N.T.; Kim, J.; Othman, M.R. Microporous ZIF-8 and ZIF-67 membranes grown on mesoporous alumina substrate for selective propylene transport. *Sep. Purif. Technol.* **2020**, *233*, 116026.

16. Kim, T.; Kim, Y.J.; Yu, C.; Kim, J.; Eum, K. Facile Fabrication of α-Alumina Hollow Fiber-Supported ZIF-8 Membrane Module and Impurity Effects on Propylene Separation Performance. *Membranes* **2022**, *12*, 1015.

17. Lian, H.; Yang, Y.; Chen, J.; Bao, B.; Yang, W.; Hou, R.; Ju, S.; Pan, Y. Highly durable ZIF-8 tubular membranes via precursor-assisted processing for propylene/propane separation. *J. Membr. Sci.* **2022**, *660*, 120813.

18. Song, E.; Wei, K.; Lian, H.; Hua, J.; Tao, H.; Wu, T.; Pan, Y.; Xing, W. Improved propylene/propane separation performance under high temperature and pressures on in-situ ligand-doped ZIF-8 membranes. *J. Membr. Sci.* **2021**, *617*, 118655.

19. Lian, H.; Bao, B.; Chen, J.; Yang, W.; Yang, Y.; Hou, R.; Ju, S.; Pan, Y. Controllable synthesis of ZIF-8 interlocked membranes for propylene/propane separation. *Sep. Purif. Technol.* **2022**, *300*, 121811.
